# Second-Order Chemometric Classification of *Candida* Species Using Excitation–Emission Matrix Fluorescence Spectroscopy: A Proof-of-Concept Study

**DOI:** 10.1021/acsomega.6c03805

**Published:** 2026-08-14

**Authors:** Lavínia H. S. Pereira, Maria R. C. Inácio, Francimara C. S. Silvestre, Rafael W. Bastos, Kássio M. G. Lima

**Affiliations:** † Biological Chemistry and Chemometrics, Institute of Chemistry, Federal University of Rio Grande do Norte, Natal, RN 5072-970, Brazil; ‡ Department of Microbiology and Parasitology, Biosciences Center, Federal University of Rio Grande do Norte, Natal, RN 5072-970, Brazil; § National Institute of Science and Technology in Human Pathogenic Fungi, Ribeirão Preto, 14040-900, Brazil

## Abstract

Microorganisms of the genus *Candida* are responsible for a wide range of fungal infections and are associated with increased morbidity and mortality rates. Although several clinical methods are available for the diagnosis of candidiasis, they have limitations related to response time and laboratory complexity. In this study, spectroscopic techniques based on excitation–emission matrix (EEM) fluorescence were explored in combination with higher-order chemometric methods, such as PARAFAC and Tucker3, coupled with classification algorithms, including Quadratic Discriminant Analysis (QDA), for the identification of seven *Candida* classes: *C. albicans*, *C. auris*, *C. glabrata*, *C. hemeulonii*, *C. krusei*, *C. parapsilosis*, and *C. tropicalis*. The analyses were conducted using a limited data set consisting of seven *Candida* species measured in ten independent replicates, resulting in 70 data points. PARAFAC-QDA and Tucker3-QDA models were developed for multiclass classification. Although evaluated on a small-scale sample size, both models reached 100% classification accuracy, sensitivity, and specificity for training and test set, highlighting the potential of multiway analysis for early exploration. The selected excitation and emission wavelengths associated with the latent factors were mainly distributed in the excitation region of 260, 270, and 280 nm, and in the emission range of 280 to 350 nm, which are strongly related to aromatic amino acids, such as tyrosine, tryptophan, and phenylalanine, reflecting the structural composition of these microorganisms. These results demonstrate the potential of the proposed approach as a proof-of-concept for the discrimination of reference *Candida* strains under controlled culture conditions, supporting future validation using independent clinical isolates.

## Graphical abstract

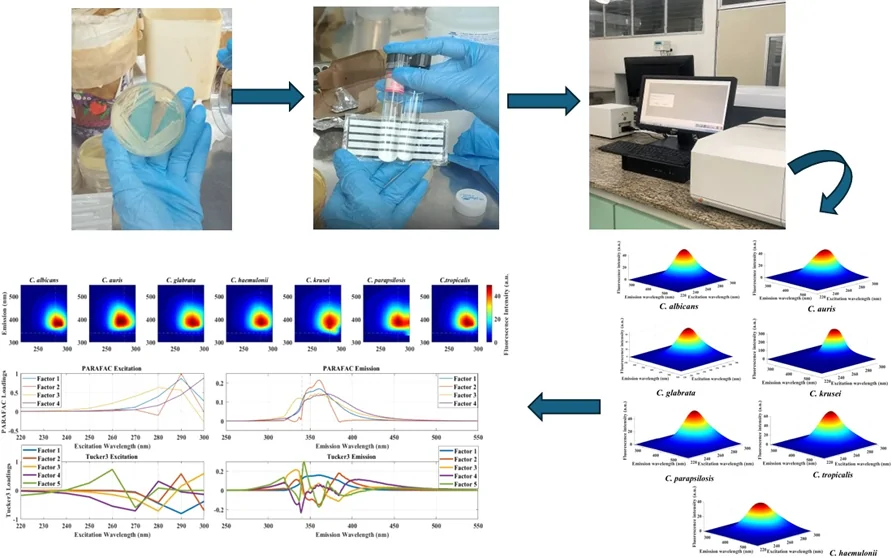

## 1 Introduction

The genus *Candida* comprises several species responsible for fungal infections, especially in immunocompromised individuals, and is associated with high morbidity and mortality rates. Although a significant proportion of candidiasis cases are attributed to *Candida albicans*, other species, such as *Candida krusei*, *Candida glabrata*, *Candida parapsilosis*, *Candida tropicalis*, *C. auris*, and *C. hemeulonii*, are also frequently isolated from clinical samples.^1–3^ Candidiasis is associated with high mortality rates and imposes a substantial financial burden on healthcare systems, potentially exceeding billions of dollars.^4–6^ Reducing mortality associated with fungal infections largely depends on the early and accurate identification of the etiological agent. Several studies demonstrate that late or incorrect diagnosis of *Candida* species is directly related to a significant increase in mortality.^7,8^ Currently, there are multiple clinical methods available for the diagnosis of candidiasis, which vary in sensitivity, specificity, response time, and laboratory complexity.

Various detection techniques, such as mass spectrometry (MS) and polymerase chain reaction (PCR), often require a prior microbiological culture step before pathogen identification. This cultivation process may take between two and 5 days from the receipt of the clinical sample (e.g., blood, catheter tip, sputum, or urine) to the acquisition of microbiological results.

This prolonged interval may delay the initiation of the most appropriate antimicrobial therapy, potentially leading to negative impacts on clinical outcomes.^9^

Hence, developing new analytical strategies that offer reduced costs and faster analysis is essential. In this context, spectroscopic methods combined with chemometric approaches have been widely employed in biological analyses as promising tools for clinical diagnosis, due to their high potential for sample classification. For instance, techniques such as ATR-FTIR and excitation–emission matrix (EEM) fluorescence have been successfully applied to the differentiation of microbial species.^10^

Excitation–emission matrix (EEM) fluorescence stands out due to its high analytical sensitivity, enabling measurements at concentrations close to those found in natural environments, in addition to being a nondestructive method. This spectroscopic approach provides a large amount of information about the analyte, allowing the direct monitoring of fluorophores, since their spectral responses are particularly sensitive to the chemical microenvironment of the sample.^11,12^ The analysis of excitation and emission wavelengths enables the identification of the composition of samples that exhibit overlapping fluorescence signals, demonstrating the applicability of the technique for the identification of biomolecules in complex systems.^13^

In this context, excitation–emission matrix (EEM) fluorescence spectroscopy may face limitations in selectivity due to significant spectral overlap and interferences from the sample matrix. However, the application of chemometric methods enables the extraction of relevant analytical information, contributing to a more robust interpretation of the data.^14^

Second-order models, such as Parallel Factor Analysis (PARAFAC), represent a generalization of Principal Component Analysis (PCA) for data sets structured as matrices.^15^ The Tucker model is a more flexible and generalized version of PARAFAC and has been widely used.^16,17^ These models are applied to the decomposition of trilinear data, providing a unique solution and enabling robust estimation of excitation and emission profiles, as well as the concentrations associated with the components present in the spectra.^18^

The decomposed data obtained from PARAFAC and Tucker models can be used to construct classification models based on Quadratic Discriminant Analysis (QDA). In addition, variable selection or dimensionality reduction through the use of latent variables may contribute to improved classification performance, although economic and technical limitations may still apply.^19^

Quadratic Discriminant Analysis (QDA), the class-conditional probability densities are modeled as multivariate normal distributions, assuming class-specific mean vectors and covariance matrices. This approach provides greater flexibility in data modeling, particularly in situations where the assumption of homogeneity of covariance matrices is not valid.^20^

However, the application of classification models may be limited by strong collinearity among variables. In this context, the use of decomposed data into latent variables, such as those obtained from PARAFAC and Tucker models, contributes to dimensionality reduction and the minimization of collinearity. Nevertheless, it is recommended that the number of samples in the training set be equal to or greater than the number of variables considered in the model, in order to ensure greater statistical robustness and stability in classification.^21^

The aim of this study was to apply excitation–emission matrix (EEM) fluorescence spectroscopy for the discrimination of seven *Candida* species (*C. albicans*, *C. krusei*, *C. glabrata*, *C. parapsilosis*, *C. tropicalis*, *C. auris*, and *C. hemeulonii*). In addition, the performance of different second-order chemometric models, including PARAFAC and Tucker, combined with Quadratic Discriminant Analysis (QDA), was evaluated and compared.

## 2 Materials and Methods

In this section, the fungal species were initially identified according to the current taxonomic nomenclature, with the corresponding previous *Candida* nomenclature indicated in parentheses. However, throughout the manuscript, the traditional *Candida* nomenclature, commonly used in clinical microbiology, was adopted to ensure consistency and readability.

### 2.1 Cultivation of Candida spp

For the differentiation of *Candida* species using excitation–emission matrix (EEM) fluorescence spectroscopy and multivariate analysis, the following strains were cultivated: *C. auris* (CBS 10923), *C. albicans* (ATCC 90029), *Nakaseomyces glabrata* (*C. glabrata*; ATCC 2001), *C. tropicalis* (51L [AMB11A]), *Pichia kudriavzevii* (*C. krusei*; AMB18L1), *C. parapsilosis* (ATCC 22019), and *C.* *hemeulonii* (CH2). The evaluated *Candida* species belong to the collection of the Department of Microbiology and Parasitology, Biosciences Center, Federal University of Rio Grande do Norte, Natal, Brazil.

For each *Candida* strain, three independent Petri dishes containing Sabouraud dextrose agar (SDA; BD DIFCO) were prepared and incubated at 37 °C for 72 h. Furthermore, each species was prepared and analyzed on different experimental days. After the incubation period, distinct isolated colonies from the culture plates of each species were collected using sterile swabs and transferred to independent, properly labeled test tubes containing liquid culture medium prior to the acquisition of the fluorescence spectra.

A total of ten independent test tubes were prepared for each *Candida* species. Subsequently, the turbidity of each suspension was adjusted to 0.5 on the McFarland scale using phosphate-buffered saline (PBS). EEM fluorescence spectroscopy analyses were then performed.

### 2.2 Excitation Emission Matrix (EEM) Fluorescence Spectroscopy

EEM fluorescence spectra of the samples were acquired using a Shimadzu RF-5301 spectrofluorometer operated with RFPC software, employing a single quartz cuvette with a volume of 3.5 mL. The monochromator slit widths were set to 3 nm for excitation and 5 nm for emission. Measurements were performed over excitation wavelengths ranging from 220 to 300 nm, at 10 nm intervals. For each excitation wavelength, emission spectra were recorded from 250 to 900 nm with a resolution of 1 nm. This protocol generated, for each sample, an EEM matrix with dimensions of 10 × 651. Although emission spectra were recorded from 250 to 900 nm, the data were truncated to the 250–550 nm region (300 variables) prior to chemometric analysis, focusing on the most informative spectral region and reducing the influence of noise and higher-order scattering effects. All EEM fluorescence spectra were preprocessed to remove Rayleigh and Raman scattering, according to the method proposed by Zepp and colleagues (2004).^22^ The algorithm removes emission peaks located at ± 10 nm around each excitation wavelength in the EEM matrix and subsequently fills the removed regions using Delaunay interpolation based on neighboring data points.^22,23^

### 2.3 Chemometric Analysis

The data obtained in this study were initially subjected to preprocessing steps and subsequently used for the construction of classification models using MATLAB R2024b software (MathWorks Inc., MA, USA). Each *Candida* species was represented by a single reference strain cultivated on three independent Petri dishes. After incubation, isolated colonies were used to prepare ten independent suspensions per species, resulting in a total of 70 samples. The samples were then divided into training (70%) and test (30%) sets using the Kennard–Stone sampling algorithm.^24^ The number of samples in the training and test sets was 49 (*n* = 49) and 21 (*n* = 21), respectively. Training (49 × 9 × 300) and test (21 × 9 × 300) matrices were generated and used for model construction and evaluation using a test set, respectively. The Kennard–Stone algorithm selects training samples based on the maximization of Euclidean distance in the variable space. Initially, the two samples with the greatest distance between them are selected. Subsequently, new samples are iteratively added by choosing the one with the largest minimum distance relative to the already selected samples. This process continues until the desired number of training samples is reached, ensuring a representative and uniform distribution of the data in the multivariate space.^25^ The PARAFAC and Tucker models applied in this study were computed using the Three-Way Weighted Discriminant Analysis toolbox (TTWD-DA 1.0). Different numbers of latent factors were systematically evaluated during the development of both PARAFAC and Tucker3 models. The estimation of the chemical ranks was initially guided by Singular Value Decomposition (SVD), combined with the assessment of spectral interpretability and classification performance.^17^ For the PARAFAC framework, although a 5-component solution was initially explored, the core consistency and factor diagnostics revealed severe mathematical redundancy, evidenced by a critical Tucker’s congruence coefficient of −0.9974 between Factors 1 and 4. Consequently, the PARAFAC model was optimized to four components to eliminate overfactoring, yielding mathematically independent and chemically meaningful loading profiles without sacrificing discriminatory power. In contrast, for the Tucker3 framework, the (5, 5, 5) unconstrained core configuration under orthogonality constraints demonstrated absolute stability, achieving mathematical convergence in only two iterations and explaining 99.85% of the total data variance. Therefore, these optimized dimensions (four factors for PARAFAC and five for Tucker3) were selected as they provide the ultimate balance among model fit, physical interpretability, and robust classification performance for the evaluated *Candida* data set.

### 2.4 PARAFAC Model

The decomposition of the three-dimensional array of trilinear matrices can be represented by eq 1

1 $$X_{\mathrm{ijk}}=\sum_{f=1}^{F}a_{\mathrm{if}}b_{\mathrm{if}}c_{\mathrm{kf}}+e_{\mathrm{ijk}}$$

where *x*_*ijk*_ represents each element of the three-dimensional data tensor *X*. The terms *a*_*if*_, *b*_*jf*_, and *c*_*kf*_ correspond to the elements of the factor matrices associated with the three modes of the trilinear model. The term *e*_*ijk*_ represents the residual error of the model. The matrices *A*, *B*, and *C* correspond, respectively, to the score matrix associated with the samples (mode 1), the loading matrix related to the excitation wavelengths (mode 2), and the loading matrix related to the emission wavelengths (mode 3).^26^

PARAFAC (Parallel Factor Analysis) is a multilinear decomposition model based on the proportionality of profiles across different data modes. The method assumes that, when a set of matrices shares common latent factors, these factors can simultaneously describe the variation present in all matrices, differing only in their weights or contribution coefficients specific to each mode.^15^

In its formulation, PARAFAC performs a trilinear approximation of the data, assuming that they can be represented by a low-dimensional structure, in which each component is described by a unique set of loading vectors. This property provides the model with an essentially unique solution, which facilitates the physicochemical interpretation of the extracted factors.^27,28^

### 2.5 Tucker3 Model

The Tucker3 model is characterized as a trilinear decomposition method originally introduced by Tucker in the mid-1960s.^29^ This model can be considered an extension of Principal Component Analysis (PCA) for higher-order or multiway data.^30^ Unlike conventional PCA, Tucker3 allows the three loading matrices to have different dimensions, enabling the number of significant components to vary across the different modes of the data set.^31^ The structural basis of the Tucker3 model is given by the matrix eq 2.

2 $$\mathrm{X̲}=\mathrm{AG}{\left(C⊗B\right)}^{t}+\mathrm{E̲}$$

In this formulation, *X* is characterized as a three-dimensional data array, decomposed into loading matrices *A (I × L)*, *B* (*J* × *M*), and *C*(*K × N*), along with a core tensor *G* (*L × M × N*). The core tensor contains elements that represent the most significant factors in the mode.^29,32^ The parameters *L*, *M*, and *N* correspond to the number of factors in the three modes of the data structure.^33^ The symbol ⊗ denotes the Kronecker product,^34^ and *E* represents the residuals.

The Tucker model constitutes a generalization of the PARAFAC model. eq 3 presents the algebraic representation of the model expressed using summation notation.

3 $$X_{\mathrm{ijk}}=\sum_{l=1}^{L}\sum_{m=1}^{M}\sum_{n=1}^{N}a_{\mathrm{il}}b_{\mathrm{jm}}c_{\mathrm{kn}}g_{\mathrm{lmn}}+e_{\mathrm{ijk}}$$

Where *x*_*ijk*_ represents the elements of the data tensor *X*; *a*_*il*_, *b*_*jm*_, and *c*_*kn*_ correspond to the elements of the loading matrices *A*, *B*, and *C*, respectively; *g*_*lmn*_ represents the elements of the core tensor *G*; and *e*_*ijk*_ corresponds to the error term associated with the model. The indices *i*, *j*, and *k* refer to the observations in the three modes of the data set.^29^

Since PARAFAC and Tucker3 models are not classification methods by themselves, the score matrix obtained after decomposition was used as input for a first-order classification algorithm.^35,36^ In this study, Quadratic Discriminant Analysis (QDA) was employed. QDA is a statistical classification method based on the Mahalanobis distance, calculated from the class-specific variance–covariance matrix.^37^ In this way, the model allows for different variance structures among classes, providing greater flexibility in group separation. The classification scores (*Q*_*ig*_) are estimated using a non-Bayesian approach, based on the covariance matrices and mean vectors of each class, to determine the membership of each sample to the most probable group^38^ (eq 4).

4 $$Q_{\mathrm{ig}}={\left(x_{i}-{\mathrm{x̲}}_{g}\right)}^{T}\Sigma_{g}^{-1}\left(x_{i}-{\mathrm{x̲}}_{g}\right)$$

Where *x*_*i*_ is defined as the Tucker3 score vector for sample *i*; *x̅*_*g*_ represents the mean vector of class *g*; and *∑*_*g*_^–1^ is the inverse of the variance–covariance matrix of class *g* ^39^

The variance–covariance matrix is calculated as shown in eq 5.^40^

5 $$\Sigma_{g}=\frac{1}{n_{g}-1}\sum_{i=1}^{n_{g}}(x_{i}-{\mathrm{x̲}}_{g}){\left(x_{i}-{\mathrm{x̲}}_{g}\right)}^{T}$$

Where *n* is the number of objects in the training set, *g* is the number of classes, and *n*_*g*_ is the number of objects in class *g* ^40^

6 $$\mathrm{CC}\left(\%\right)=100-\frac{\varepsilon_{1}\varepsilon_{2}}{N}\times 100$$

7 $$\mathrm{AC}\;\left(\%\right)=\frac{\mathrm{TP}+\mathrm{TN}}{\mathrm{TP}+\mathrm{TN}+\mathrm{FP}+\mathrm{FN}}\times 100$$

8 $$S\;\left(\%\right)=\frac{\mathrm{TP}}{\mathrm{TP}+\mathrm{FN}}\times 100$$

9 $$\mathrm{SP}\;\left(\%\right)=\frac{\mathrm{TN}}{\mathrm{TN}+\mathrm{FP}}\times 100$$

10 $$F‐\mathrm{Score}\;\left(\%\right)=\frac{2\times \mathrm{SENS}\times \mathrm{SPEC}}{\mathrm{SENS}+\mathrm{SPEC}}$$

11 $$G‐\mathrm{Score}\;\left(\%\right)=\sqrt{\mathrm{SENS}\times \mathrm{SPEC}}$$

In the equations, TP, TN, FP, and FN represent true positives, true negatives, false positives, and false negatives, respectively. The correct classification rate (CC%) corresponds to the percentage of samples correctly classified in the training and test sets, considering their true class membership, where ε_1_ and ε_2_ are the errors associated with classes 1 and 2, respectively.^41,42^

Accuracy (AC), also referred to as the prediction rate, indicates the overall proportion of correctly classified samples by the model, considering both true positives and true negatives. Sensitivity (*S*) represents the proportion of correctly classified positive samples, while specificity (SP) corresponds to the fraction of correctly identified negative samples.

The F-score corresponds to the harmonic mean of sensitivity and precision and is widely used to evaluate model performance in imbalanced data sets. In turn, the G-score reflects the balance of the model’s performance between positive and negative classes, regardless of class distribution.^41–44^

## 3 Results and Discussion

For each sample, a total fluorescence matrix (EEM) was constructed from the systematic variation of excitation and emission wavelengths. Thus, each EEM obtained contains relevant information related to each fungal class. The main spectral characteristics of the samples used in the present study can be observed in Figure 1, which presents the excitation/emission fluorescence contour plots of the seven analyzed species: *C. albicans* (ATCC 90029) (Figure 1A), *C. auris* (CBS 10923) (Figure 1B), *C. glabrata* (ATCC 2001) (Figure 1C), *C. krusei* (AMB18L1) (Figure 1D), *C. parapsilosis* (ATCC 22019) (Figure 1E), *C. tropicalis* (51L (AMB11A)) (Figure 1F), and *C. hemeulonii* (CH2) (Figure 1G), after removal of Rayleigh and Raman scattering.

**Figure 1:**
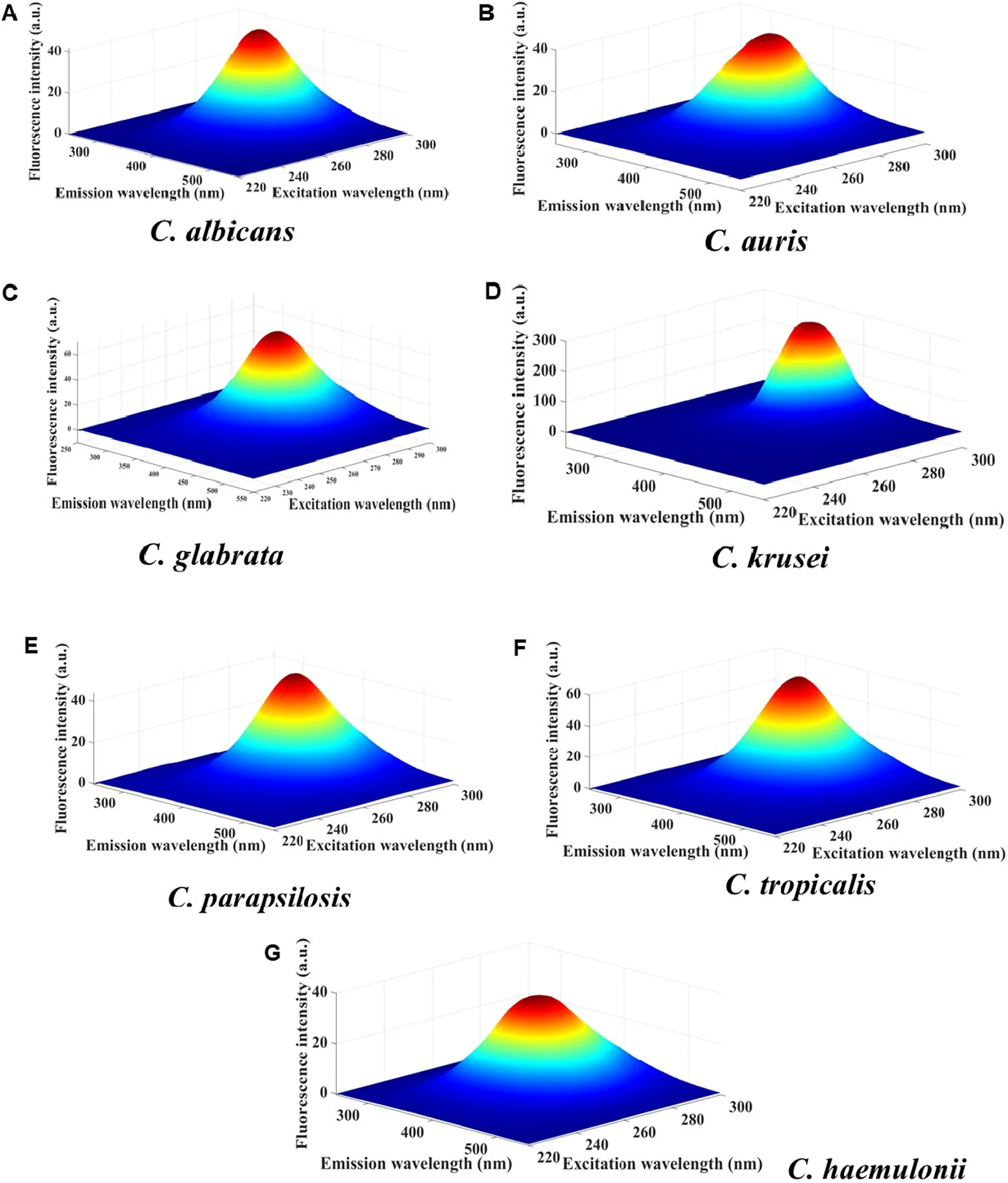
Emission and excitation matrix *C. albicans* (A), *C. auris* (B), *C. glabrata* (C), *C. krusei* (D), *C. parapsilosis* (E), *C. tropicalis* (F) and *C. hemeulonii* (G).

The three-dimensional excitation–emission matrix (EEM) fluorescence plots present the average spectra of samples from seven *Candida* species, highlighting differences in fluorescence intensities among them (Figure 1). The spectra were previously preprocessed to remove Rayleigh and Raman scattering.

Among the species, a distinct behavior is observed for *C. krusei* (Figure 1D), which stands out by exhibiting a significantly higher fluorescence peak, reaching values close to 300 au This may stem from a higher concentration of intracellular fluorophores, such as proteins or fluorescent coenzymes, or differences in cellular metabolism under the experimental conditions.^45^

In contrast, *C. albicans* (Figure 1A), *C. auris* (Figure 1B), *C. hemeulonii* (Figure 1G), and *C. parapsilosis* (Figure 1E) exhibited moderate intensity peaks, ranging from 35 to 45 au, while *C. glabrata* (Figure 1C) and *C. tropicalis* (Figure 1F) showed intermediate intensity values around 60 au These variations in intrinsic fluorescence reinforce the potential of this technique as a discriminative parameter for taxonomic differentiation within the genus.

In all species, a prominent excitation band around 280 nm with corresponding emission is observed, which is characteristic of tryptophan fluorescence. This signal is particularly intense in *C. krusei* (Figure 1D), reaching approximately 300 au The excitation region between 270 and 275 nm, with emission between 300 and 320 nm, is associated with tyrosine, whose spectral band may vary in width depending on the species. The three aromatic amino acids, tyrosine, tryptophan, and phenylalanine present excitation maxima that correspond to the selected factor peaks at 260, 270, and 280 nm, respectively, as well as emission maxima around 280, 300, and 350 nm.^46^

Although subtle differences can be observed among the spectral profiles of the seven classes, these variations are not sufficiently discriminative, since the spectra correspond to averages of individual measurements. When analyzed together, the profiles exhibit a high degree of overlap and similarity, rendering direct discrimination via visual inspection unfeasible.^47^

In this context, multivariate analysis techniques were applied using PARAFAC-QDA and Tucker3-QDA models to extract the maximum relevant information from the data and highlight subtle variations not evident in the raw spectral profiles, enabling improved class discrimination.^25,48^ The performance of the models was evaluated using the following figures of merit: accuracy, sensitivity, specificity, F-score, and G-score.

### 3.1 EEMs Modeling

The excitation–emission fluorescence matrices (EEMs) were organized into a three-dimensional array (fluorescence cube), obtained by stacking the individual matrices into a single data set. Multivariate models, namely PARAFAC and Tucker, were applied to this array using five latent factors, which explained 100% of the observed variance. For all models, the loading vectors, sample scores, and canonical scores were evaluated, allowing both spectral interpretation and assessment of discriminant capability.

The discrimination among the seven *Candida* species was performed by combining the PARAFAC and Tucker models with Quadratic Discriminant Analysis (QDA), which was directly applied for sample classification.

### 3.2 Scores PARAFAC and Tucker3—QDA

Figure 2 shows the samples projected onto the three-dimensional (3D) discriminant space obtained from the optimized scores of the PARAFAC model with four components (Figure 2A) and the Tucker3 model with five components (Figure 2B), both combined with Quadratic Discriminant Analysis (QDA). Each point represents an individual *Candida* sample, where the training and test set are evaluated simultaneously. In this multiway discriminant space, the spatial distance between the clusters directly reflects the degree of chemical and biological separation achieved between the fungal species.

**Figure 2:**
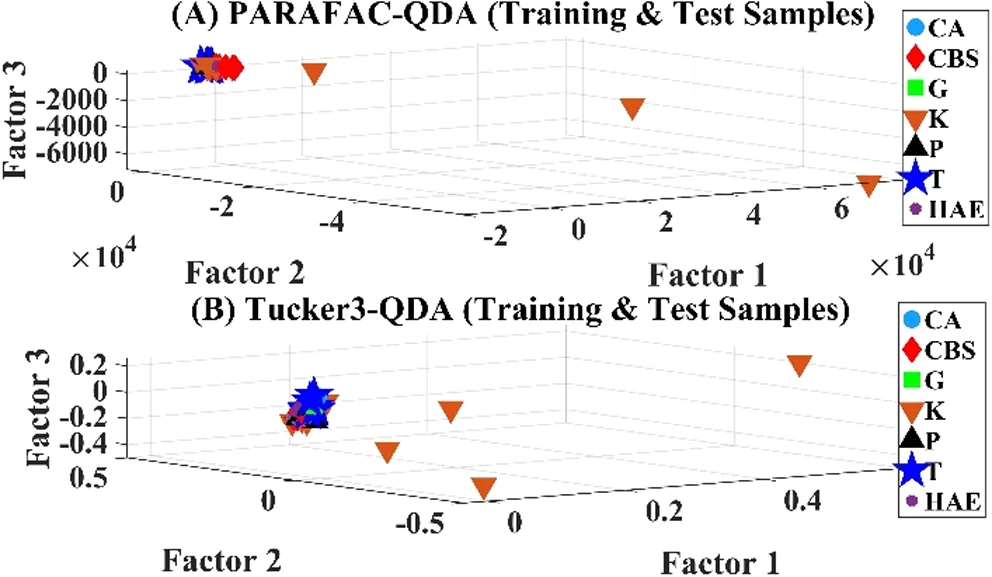
Three-dimensional (3D) scores space for PARAFAC-QDA computed with four components (A) and Tucker3-QDA computed with five components (B) for seven *Candida* species (CA, CBS, G, K, AP, T, and HAE) evaluated across training and test set.

In Figure 2A (3D PARAFAC–QDA), a well-defined and robust sample clustering according to each *Candida* species is observed, demonstrating that the optimized 4 component model successfully captures the core chemical variance from the fluorescence spectral profiles. *C. krusei* (K) stands out significantly, appearing partially isolated from the other species in the three-dimensional discriminant space. This clear separation is highly consistent with its unique and intense fluorescence fingerprint profile shown in Figure 1D. In contrast, the remaining species form tighter, distinct groups that reflect their biological and spectral relationships under the optimized trilinear system.

In Figure 2B (3D Tucker3–QDA), an equally powerful and complementary discrimination performance is achieved. The flexibility of the unconstrained core (5, 5, 5) combined with the quadratic discriminant boundaries allows for an exceptional representation of the multiway interactions among the samples. This architecture minimizes internal class variance, leading to highly compact clusters for species such as *C. albicans* (CA), *C. auris* (CBS), *C. glabrata* (G), *C. parapsilosis* (P), *C. tropicalis* (T), and *C. hemeulonii* (HAE). The performance observed for the test set suggests that the proposed models were able to correctly classify samples not used during model calibration. However, because both training and test samples originated from the same reference strains, these results should not be interpreted as independent external validation.

### 3.3 Loadings

The multiway loading plots obtained from the PARAFAC–QDA and Tucker3–QDA decompositions, integrated alongside excitation–emission landscapes (Figure 3), allow a comprehensive evaluation of the relative contribution of each spectral variable to the extracted latent factors. The analysis of these loading profiles highlights the most relevant excitation and emission wavelengths responsible for the discrimination of the seven Candida classes. For the Tucker3 model, the highest explained variance was achieved using five latent factors (Figure 3, bottom row), while the PARAFAC model successfully resolved the underlying chemical variance using four components (Figure 3, middle row).

**Figure 3:**
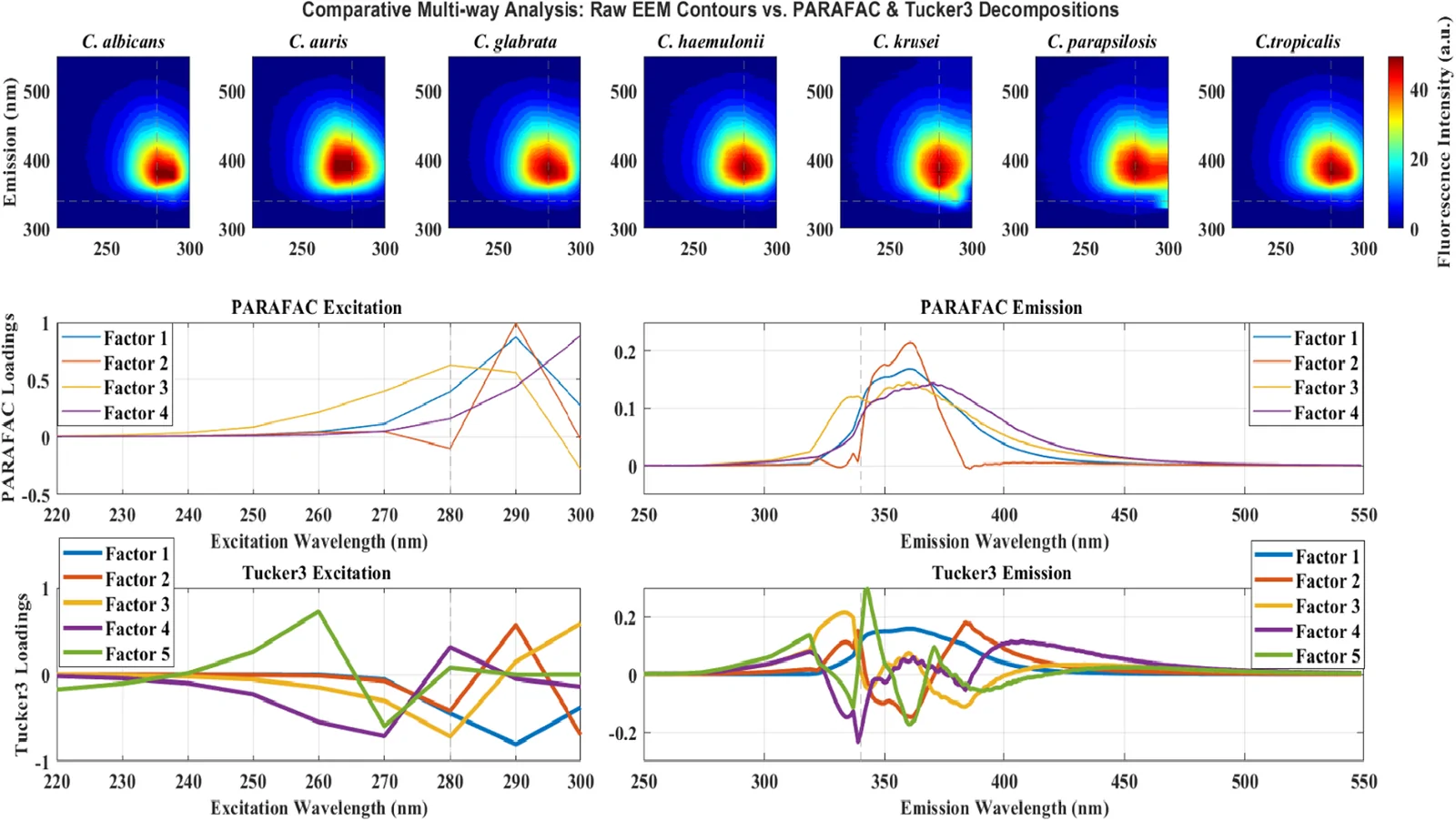
Integrated multiway analysis of the seven *Candida* species. Top row: Raw three-dimensional excitation–emission matrix (EEM) fluorescence contour landscapes. Middle row: Excitation and emission loading profiles resolved by the 4-component PARAFAC model. Bottom row: Excitation and emission loading profiles resolved by the 5-component Tucker3 model. Dashed gray lines indicate the spectral cross-sectional correspondence of peak maxima (Δλexc = 280 nm and Δλem = 340 nm).

The specific wavelengths identified in the loading profiles can be interpreted as the direct signatures of specific endogenous fluorophores.^49^ In the PARAFAC framework, the emission loadings represent reconstructed, pure spectral profiles associated with the intrinsic fluorescence contributions of these cellular components.^50^ In the excitation domain, the main spectral contributions driving the models were observed at 260, 270, 280, and 290 nm. In the emission domain, the PARAFAC profiles highlighted significant features at 320, 348, 360, 361, 374, and 391 nm, whereas the orthogonal Tucker3 profiles captured critical shape variations and coordinate transitions at 339, 343, 360, 362, 371, 383, 384, and 403 nm.

The selected spectral region includes key fluorescence signals primarily associated with aromatic amino acids, which are highly relevant for the discrimination of *Candida* species. These spectral regions are intrinsically linked to endogenous fluorophores such as NAD­(P)­H, flavins, and structural proteins, directly reflecting the metabolic activity and macromolecular composition of the different Candida cell walls and matrices.^51,52^ Additionally, the resolved excitation and emission ranges are consistent with the characteristic fluorescence of aromatic amino acids such as tyrosine, tryptophan, and phenylalanine which typically present excitation maxima around 260–280 nm and emission envelopes in the range of 280–350 nm.^46^

Despite these clear mathematical boundaries extracted by the multiway algorithms, direct visual inspection of the raw, individual spectral profiles remains insufficient for a reliable and reproducible discrimination. This limitation arises because the raw EEM data represent averaged macroscopic responses and exhibit a high degree of overlapping signals among the seven *Candida* classes (Figure 3, top row), underscoring the absolute necessity of coupling multiway decomposition with supervised discriminant analysis (QDA).

After further validation with a larger data set and a deeper understanding of the biochemical origin of these spectral signatures, synchronous fluorescence spectroscopy may be explored as a complementary screening approach due to its higher speed and selectivity. In this technique, excitation and emission wavelengths are scanned simultaneously while maintaining a constant wavelength interval (Δλ), known as the Stokes shift. The effectiveness of this alternative approach heavily depends on the appropriate selection of (Δλ), which requires the exact prior knowledge of the characteristic spectral signatures of the analyzed species provided by the PARAFAC and Tucker3 models developed in this work.^52,53^

### 3.4 PARAFAC-QDA/Tucker3-QDA Canonical Scores

In order to evaluate and validate the spectral discrimination capability among the seven fungus species of the *Candida* genus (*C. albicans*, *C. auris*, *C. glabrata*, *C. krusei*, *C. parapsilosis*, *C. tropicalis*, *and* *C.* *hemeulonii*), the excitation–emission matrix (EEM) data were submitted to PARAFAC–QDA and TUCKER3–QDA multiway modeling. This multivariate classification approach allowed correlating the spectral profiles and fingerprints obtained for each fungal isolate with their respective real microbiological identities. The results of this modeling and the analytical robustness achieved by the quadratic classifiers are graphically consolidated in Figure 4 and quantified in detail by the confusion matrices presented in Table 2.

**Figure 4:**
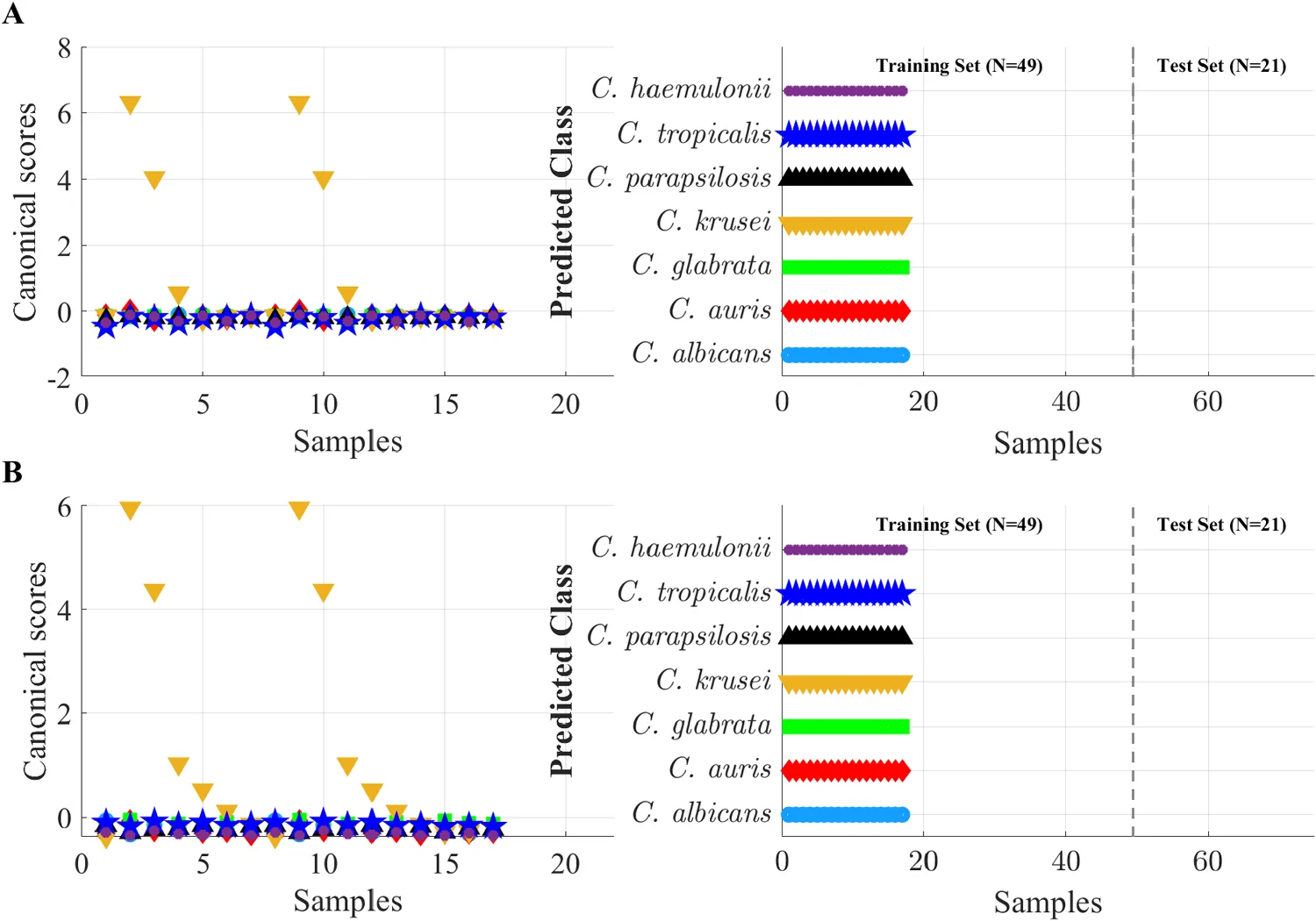
Multivariate classification results via quadratic discriminant modeling for (A) PARAFAC–QDA and (B) TUCKER3–QDA frameworks. The plots on the left display the canonical scores obtained for the test set (*N* = 21). The plots on the right illustrate the predicted classes for all analyzed samples, where the vertical dashed line at 49.5 delimits the partition between the training set (*N* = 49) and the test set (*N* = 21).

Figure 4 presents the overall graphic performance obtained for both models. On the left (Figure 4A and B) are the canonical scores plots for the test set, revealing the efficient projection of the samples into an optimized latent space that maximizes interspecies separation. On the right, the predicted class plots visually demonstrate the assignment of each sample to its respective diagnostic class (from 1 to 7). The vertical dashed line established at 49.5 rigidly delimits the division between the training set (*N* = 49) and the test set (*N* = 21), showcasing the high accuracy rate in both domains.

To quantify and consolidate the analytical efficiency of this classification in detail, Table 1 presents the Confusion Matrices for the training and test set stages of both models. The mathematical analysis of the matrices ratifies and complements the visual findings of the graphic modeling. The values arranged along the main diagonals of each matrix indicate the number of samples correctly classified into their real biological categories.

**Table 1:** Confusion Matrix for the Classification of Seven *Candida* Species using PARAFAC–QDA and Tucker3–QDA Models, Stratified by Training, Validation, and Test Set

	*C. albicans*	*C. auris*	*C. glabrata*	*C. hemeulonii*	*C. krusei*	*C. parapsilosis*	*C. tropicalis*	Accuracy (%)
PARAFAC–QDA Framework								
Training Set (*N* = 49)	7	7	7	7	7	7	7	100%
Validation Set (*N* = 49)	7	7	7	7	7	7	7	100%
Test Set (*N* = 21)	3	3	3	3	3	3	3	100%
TUCKER3–QDA Framework								
Training Set (*N* = 49)	7	7	7	7	7	7	7	100%
Validation Set (*N* = 49)	7	7	7	7	7	7	7	100%
Test Set (*N* = 21)	3	3	3	3	3	3	3	100%

The predicted class plots (right graphic) indicate that all samples were correctly classified, demonstrating that both PARAFAC–QDA and Tucker3–QDA achieved high classification performance. These results highlight the potential of combining EEM fluorescence spectroscopy with second-order chemometric methods for the identification and classification of microbial species.

It can be observed in Table 2 that the true-positive rates for the training set resulted in an expressive analytical accuracy, indicating that the calibration stage of the quadratic classifiers robustly captured the spectral fingerprint profiles of each species. More importantly, the confusion matrix results for the test set (*N* = 21), with 3 independent samples per species indicate a high classification performance within the limitations of the current experimental design, suggesting a preliminary indication of model stability that remains to be confirmed with independent external biological isolates.

**Table 2:** Classification Performance Metrics for PARAFAC-QDA and Tucker3-QDA Models^a^

	Class 1	Class 2	Class 3	Class 4	Class 5	Class 6	Class 7
Training (%)	100	100	100	100	100	100	100
Validation (%)	100	100	100	100	100	100	100
Test (%)	100	100	100	100	100	100	100
Accuracy (%)	100	100	100	100	100	100	100
Sensitivity (%)	100	100	100	100	100	100	100
Specificity (%)	100	100	100	100	100	100	100
F-Score	100	100	100	100	100	100	100

a Identical values were obtained for both models.

From these confusion matrices, crucial analytical figures of merit such as Sensitivity and Specificity were derived for each class, which are also summarized in Table 2. Both multivariate classification models displayed ideal indices (or very close to 100%), proving that the proposed methodologies represent highly selective, rapid, and reliable alternatives for the automated microbiological identification of critical *Candida* species.

## 4 Figures of Merit

The figures of merit obtained for both PARAFAC–QDA and Tucker3–QDA models showed maximum performance, with accuracy, sensitivity, specificity, F-score, and G-score reaching 100% for all evaluated classes, indicating complete correct classification of the samples.

Despite these results, the analysis of canonical score plots revealed that the PARAFAC–QDA model exhibited partial overlap between some classes, suggesting that class separation in the latent space was not fully achieved, possibly due to the trilinear constraint of the model. In contrast, the Tucker3–QDA model showed a more consistent distribution of samples, indicating a greater ability to capture structural variations in the data.

It is important to note that the perfect classification performance may be influenced by factors such as data set size, intrinsic class separability, or potential overfitting. Therefore, additional validation strategies are recommended to assess model robustness and generalization.

Overall, the results demonstrate the potential of combining EEM fluorescence spectroscopy with second-order chemometric methods for the discrimination of the *Candida* reference strains evaluated in this study, with species, Tucker3–QDA showing slightly better consistency in the latent variable space.

It is important to note that the perfect classification performance may be influenced by factors such as data set size, intrinsic class separability, or potential overfitting. Therefore, additional validation strategies are recommended to assess model robustness and generalization. Furthermore, because each species was represented by a single reference strain and the training and test sets were generated from samples derived from the same strains, the reported performance may partially reflect strain-specific spectral characteristics rather than complete species-level discrimination. Therefore, the results should be interpreted as a proof of concept demonstration, and additional validation using independent isolates, external data sets, different culture conditions, and clinically relevant sample matrices will be necessary to assess the robustness and generalizability of the proposed methodology.

Compared to conventional identification methods such as biochemical profiling, PCR, MALDI-TOF MS, and ATR-FTIR spectroscopy, the proposed EEM fluorescence approach offers the advantages of rapid spectral acquisition and the generation of multidimensional fluorophore information that can be effectively exploited through chemometric modeling. However, unlike direct molecular approaches, the current workflow still relies on prior microbial cultivation, inoculum standardization, and controlled spectral acquisition. Therefore, the potential gain in analytical time is primarily associated with the postculture identification step rather than the clinical diagnostic process as a whole. Additionally, further studies involving independent isolates, clinically relevant sample matrices, external validation, and direct comparisons with established identification techniques are required to evaluate the practical advantages, robustness, and clinical applicability of the proposed methodology. Since the present study focused exclusively on pure cultures under controlled conditions, validation of the methodology in mixed cultures or contaminated samples was not performed. Likewise, assessing the robustness of the method against variations in culture media, incubation times, inoculum densities, and strain variability constitutes an essential perspective for future work.

The implementation of the proposed methodology in routine clinical laboratories would require access to EEM fluorescence instrumentation and chemometric data processing tools. Although spectral acquisition is relatively fast following sample preparation, the development, validation, and maintenance of the models currently demand specialized expertise in multivariate data analysis. Future developments focusing on automation and software interfaces could facilitate its translation into routine clinical practice.

## Conclusions

This study demonstrated the potential of excitation–emission matrix (EEM) fluorescence spectroscopy combined with second-order multivariate classification models for the discrimination of *Candida* species cultivated under controlled experimental conditions. Higher-order classification frameworks, specifically PARAFAC–QDA and Tucker3–QDA, were successfully applied and exhibited satisfactory performance for the analyzed data set.

The most relevant excitation and emission wavelengths were primarily associated with aromatic amino acids, such as tyrosine, tryptophan, and phenylalanine, suggesting their potential contribution to the spectroscopic differentiation of the evaluated fungal species.

The proposed approach demonstrates the potential of excitation–emission matrix (EEM) fluorescence spectroscopy combined with multiway chemometric analysis for the discrimination of *Candida* reference strains cultivated under controlled laboratory conditions. The present work should be interpreted as a culture-based proof-of-concept study, given that only reference strains were evaluated and the training and test sets were generated from samples derived from the same strains. Although the approach offers advantages such as rapid spectral acquisition, nondestructive measurements, and relatively low operational costs, its broader applicability remains to be established through studies involving independent biological isolates, external data sets, and clinically relevant samples.

Therefore, future studies including larger data sets, independent biological isolates, mixed cultures, and clinically relevant sample matrices will be required to better assess the robustness, reproducibility, and applicability of the proposed methodology for routine diagnostic applications.
